# Exploring the pathogenesis and immune infiltration in dilated cardiomyopathy complicated with atrial fibrillation by bioinformatics analysis

**DOI:** 10.3389/fimmu.2023.1049351

**Published:** 2023-01-17

**Authors:** Ting Gan, Jing Hu, Anwer Khalid Okab Aledan, Wenhu Liu, Cui Li, Shuai Lu, Ya Wang, Qian Xu, Yan Wang, Zhaohui Wang

**Affiliations:** ^1^ Department of Cardiology, Union Hospital, Tongji Medical College, Huazhong University of Science and Technology, Wuhan, China; ^2^ Hubei Key Laboratory of Biological Targeted Therapy, Union Hospital, Tongji Medical College, Huazhong University of Science and Technology, Wuhan, China; ^3^ Hubei Provincial Engineering Research Center of Immunological Diagnosis and Therapy for Cardiovascular Diseases, Union Hospital, Tongji Medical College, Huazhong University of Science and Technology, Wuhan, China; ^4^ Department of Infectious Diseases, Union Hospital, Tongji Medical College, Huazhong University of Science and Technology, Wuhan, China; ^5^ Department of Cardiovascular Surgery, Union Hospital, Tongji Medical College, Huazhong University of Science and Technology, Wuhan, China

**Keywords:** dilated cardiomyopathy, atrial fibrillation, bioinformatics, differentially expressed genes, hub immune-related genes, immune infiltration

## Abstract

**Background:**

Atrial fibrillation (AF) is a serious complication of dilated cardiomyopathy (DCM), which increases the risk of thromboembolic events and sudden death in DCM patients. However, the common mechanism of DCM combined with AF remains unclear. This study aims to explore the molecular mechanism and analyze immune infiltration in DCM complicated with AF through comprehensive bioinformatics analysis.

**Methods:**

The gene expression datasets of DCM (GSE141910) and AF (GSE41177 and GSE79768) were obtained from the Gene Expression Omnibus database. Gene enrichment analyses were performed after screening the common differentially expressed genes (DEGs) of DCM and AF. Protein-protein interaction network was constructed in the STRING database and visualized in Cytoscape software, which helped to further screen the central functional modules of DEGs and hub genes. In addition, ImmuCellAI algorithm was performed to estimate immune infiltration patterns, and Spearman correlation was conducted to investigate the correlation between the abundance of multiple immune cells and the expression levels of hub immune-related genes after obtaining hub immune-related genes from the ImmPort database. The hub immune-related genes expression and immune infiltration patterns were additionally verified in the validation datasets (GSE57338, GSE115574, and GSE31821). The diagnostic effectiveness of hub immune-related genes was evaluated through Receiver Operator Characteristic Curve analysis.

**Results:**

A total of 184 common DEGs in DCM and AF were identified for subsequent analyses. The functions of hub genes were significantly associated with immune responses. We identified 7 hub immune-related genes (*HLA-DRA*, *LCK*, *ITK*, *CD48*, *CD247*, *CD3D*, and *IL2RG*) and a spectrum of immune cell subsets including Monocyte, Neutrophil, and follicular helper T (Tfh) cells were found to be concurrently dysregulated in both DCM and AF. 7 hub immune-related genes were predominantly favorably correlated with Tfh cells and were primarily negatively correlated with Neutrophil infiltrations in DCM and AF. *CD48*+*CD3D* were verified to diagnose DCM and AF with excellent sensitivity and specificity, showing favorable diagnostic value.

**Conclusions:**

Our study reveals that immune cells (Tfh cells) disorders caused by hub immune-related genes (*CD48* and *CD3D*) may be the common pathogenesis of DCM combined with AF, which lays a foundation for further immune mechanism research.

## Introduction

Dilated cardiomyopathy (DCM) is defined as left ventricular or biventricular systolic and dilation dysfunction without coronary artery disease or abnormal load proportional to the degree of left ventricular damage (1). DCM is one of the most common causes of heart failure (HF) and the most frequent indication for cardiac transplantation (2). Atrial fibrillation (AF) is a frequently sustained arrhythmia in DCM, and they often complicate each other, jointly increasing the risk of stroke and all-cause mortality, and seriously jeopardizing cardiovascular health (3). Genetic variation, insufficient knowledge of the underlying pathophysiology, under-diagnosis of AF in DCM, and thromboembolic disease due to AF are considered to be the main causes of poor prognosis. Therefore, it is urgent to explore the potential molecular mechanism of the co-pathogenesis of DCM and AF.

From a pathophysiological perspective, DCM contributes to an increased risk of AF through several mechanisms, including excessive atrial pressure, maladaptive gene expression, abnormal myocardial conduction, and cardiac structural remodeling (4). Elevated atrial pressure and atrial dilatation in DCM patients promote myocardial fibrosis and scar formation, which eventually conduce to conduction abnormalities and the occurrence and development of AF (4). Furthermore, inflammation and immune responses are also common features of many DCM and AF (5).

Common transcriptional characteristics may offer a new way of thinking for the co-pathogenesis of DCM and AF. Our study aimed to identify the hub genes associated with the pathogenesis of DCM complicated with AF. We comprehensively analyzed three datasets (GSE141910, GSE41177, and GSE79768) obtained from the Gene Expression Omnibus (GEO) database. A combination of bioinformatics and gene enrichment analyses was applied to identify common differentially expressed genes (DEGs) of DCM and AF and explore the shared signaling pathways of DEGs, which initially revealed the potential molecular mechanisms. Subsequently, protein-protein interaction (PPI) network was established using the STRING database, and the central functional modules of DEGs and hub genes were identified by Cytoscape software. Moreover, we identified seven important hub immune-related genes and analyzed the landscape of immune cell infiltration in DCM and AF by ImmPort database and ImmuCellAI. In addition, we further analyzed the relevance between the abundance of immune cells and the expression levels of hub immune-related genes. Finally, we verified the trend of hub immune-related gene expression and their correlation with immune cell infiltration in the independent validation datasets (GSE57338, GSE115574, and GSE31821). Receiver Operator Characteristic Curve (ROC) analysis was applied to predict the diagnostic effectiveness of hub immune-related genes. The hub immune-related genes identified here between DCM and AF and the immune cell disorders caused by them are expected to lay the foundation for the study of the pathogenesis of these two diseases.

## Methods

### Data acquisition and pre-processing

The GSE141910, GSE41177, and GSE79768 gene expression datasets were obtained from the GEO database(https://www.ncbi.nlm.nih.gov/geo/) (6). The dataset GSE141910 was acquired using the GPL16791 Illumina HiSeq 2500 (Homo sapiens) from a cohort comprised of 366 samples, including left ventricle tissue samples from 166 DCM patients, 28 Hypertrophic cardiomyopathy (HCM) patients, 6 Peripartum cardiomyopathy (PPCM) patients, and 166 healthy controls. Only healthy samples and DCM samples were included for bioinformatics analysis in this study. The datasets GSE41177 and GSE79768 were acquired using the GPL570 Affymetrix Human Genome U133 Plus 2.0 Array. The dataset GSE41177 included left atrial-pulmonary vein and left atrial appendage tissue samples from 3 sinus rhythm patients and 16 atrial fibrillation patients. The dataset GSE79768 included left atrial and right atrial tissue samples from 6 sinus rhythm patients and 7 atrial fibrillation patients. Only left atrial tissue samples were included for bioinformatics analysis in this study. R software (R 4.2.0) was used to analyze the obtained datasets. The probes are converted into gene symbols using platform annotation profiles. Probes without corresponding gene symbols were eliminated, and the average value of multiple probes of a gene was taken. The abnormal samples were removed by hierarchical clustering analysis. After merging datasets (GSE41177 and GSE79768), the R package “sva” was used to eliminate heterogeneity due to different batches and platforms (7).

### Identification of DEGs

The R package “limma” was applied to identify DEGs between control groups and diseased groups (8). By Benjamini and Hochberg method to adjust *P*-values, only the genes with adjusted *P*-value < 0.05 and |logFC (fold change) | > 0.6 were identified as DEGs. The common DEGs in DCM and AF were obtained by the online Venn diagram tool.

### Enrichment analyses of DEGs

The R package “clusterProfiler” (9) was used to perform Gene Ontology analysis (GO) and Kyoto Encyclopedia of Genes and Genomes (KEGG) pathway enrichment analysis to investigate the biological functions of DEGs. Adjusted *P*-values < 0.05 were considered significant. The results were displayed by the R package “ggplot2”.

### Protein-protein interaction network construction and module analysis

The PPI network of DEGs with a filtering condition (score>0.4) was constructed by STRING (https://string-db.org) (10) and visualized by Cytoscape (version 3.9.1, http://www.cytoscape.org) (11). The plugin molecular complex detection technology (MCODE) of Cytoscape was utilized to identify central functional modules. Filtering criteria were performed with default values. Then the R package “clusterProfiler” were applied to perform GO and KEGG enrichment analysis of the module genes involved.

### Identification and enrichment analysis of hub genes

The plugin cytoHubba in Cytoscape was utilized to score each node gene by 7 algorithms, including MNC (Maximum Neighborhood Component), Degree, EcCentricity, EPC (Edge Percolated Component), Closeness, MCC (Maximal Clique Centrality), and Radiality. The R package “UpSet” was conducted to identify hub genes in the top 30 node genes scored by each algorithm. GeneMANIA (http://www.genemania.org) (12) was devoted to establishing a co-expression network for these hub genes, which allows the prediction of gene interactions and analysis of gene function.

### Hub immune-related genes in DCM and AF

A list of immune-related genes was downloaded from the ImmPort database (https://www.immport.org) (13), and hub immune-related genes were obtained by intersecting immune-related genes with hub genes by drawing a Venn diagram.

### Immune infiltration analyses

The Immune Cell Abundance Identifier (ImmuCellAI, http://bioinfo.life.hust.edu.cn/web/ImmuCellAI) (14) is an immune infiltration scoring tool that provides a comprehensive prediction for the ratio of 24 immune cell types based on the gene expression data, including 18 T-cell subtypes and 6 other immune cells (macrophages, B cells, dendritic cells, natural killer cells, neutrophils, and monocytes). Wilcoxon rank-sum test was used to calculate differences in immune cell abundance between diseased and control groups. Spearman correlation was applied to investigate the relevance between the abundance of immune cells and the expression levels of hub immune-related genes. The results of immune infiltration analyses were visualized by the R packages “reshape2”, “ggplot2”, “dplyr”, and “ggpubr”.

### Validation of hub immune-related genes and immune infiltration

The expressions of the hub immune-related genes were extracted from independent datasets (DCM: GSE57338; AF: GSE115574 and GSE31821). The differences in the expressions of these hub immune-related genes between diseased and control samples were calculated by Wilcoxon rank-sum test and visualized by R package “ggplot2” and “ggpurb”. Similarly, immune cell abundance in the validation datasets was predicted by ImmuCellAI and the correlation between the abundance of immune cells and the expression levels of hub immune-related genes was investigated by Spearman correlation. We then obtained dataset (GSE86569) of AF without HF and AF with HF to analyze the immune infiltration patterns and verify the expression of immune-related genes (*CD48* and *CD3D*). *P*-values < 0.05 were considered significant.

### Analysis of the predictive value of hub immune-related genes

The R package “pROC” was applied to ROC analysis to predict the diagnostic effectiveness of hub immune-related genes. The area under the ROC curve (AUC) value was applied to estimate the diagnostic validity in differentiating DCM and AF from control samples in the training datasets (GSE141910, GSE41177, and GSE79768) and validation datasets (GSE57338, GSE115574, and GSE31821).

## Results

### Identification of DEGs from patients with DCM and AF

**Figure 1:**
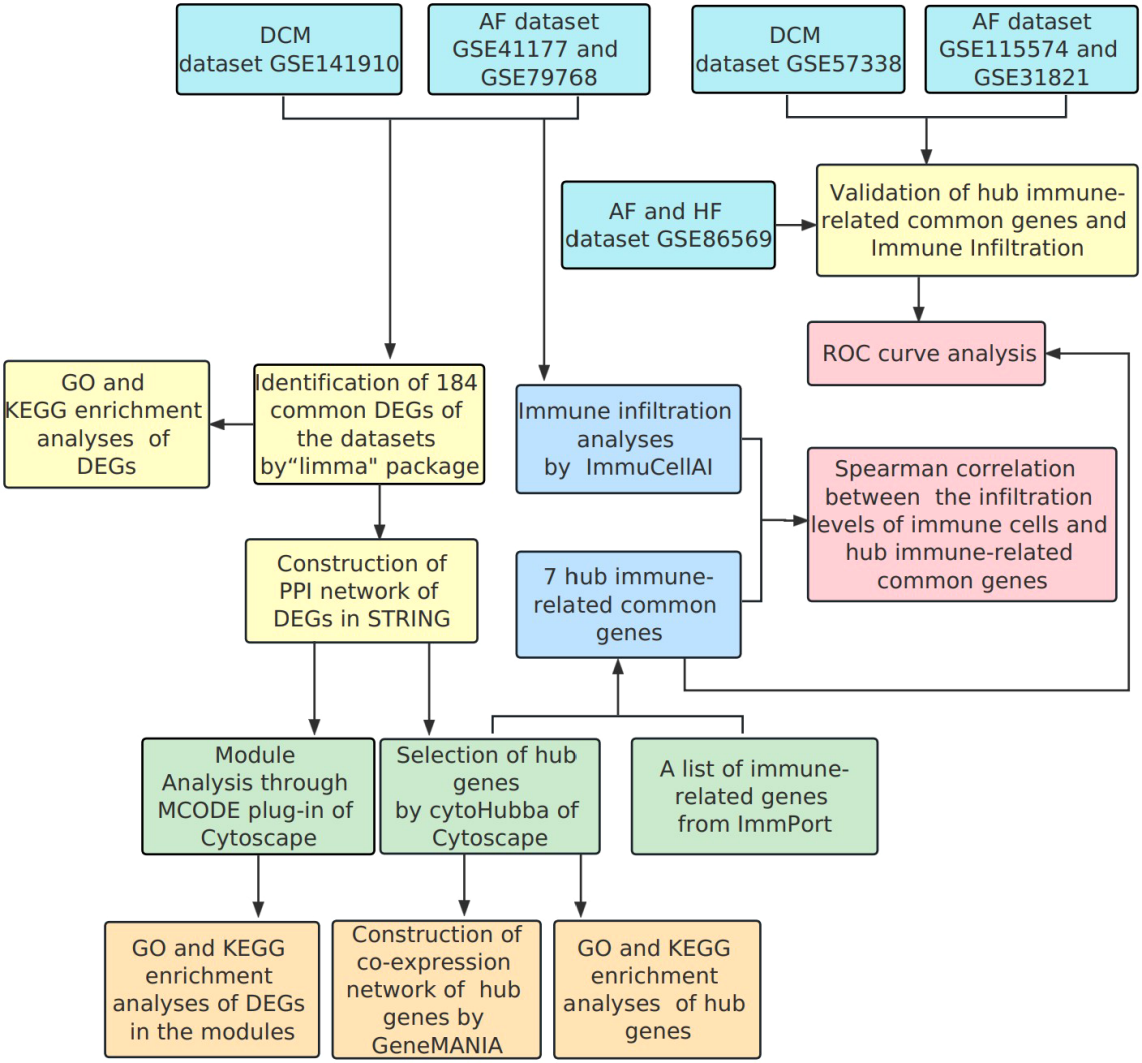
Flowchart of the bioinformatics data analysis.

**Figure 2:**
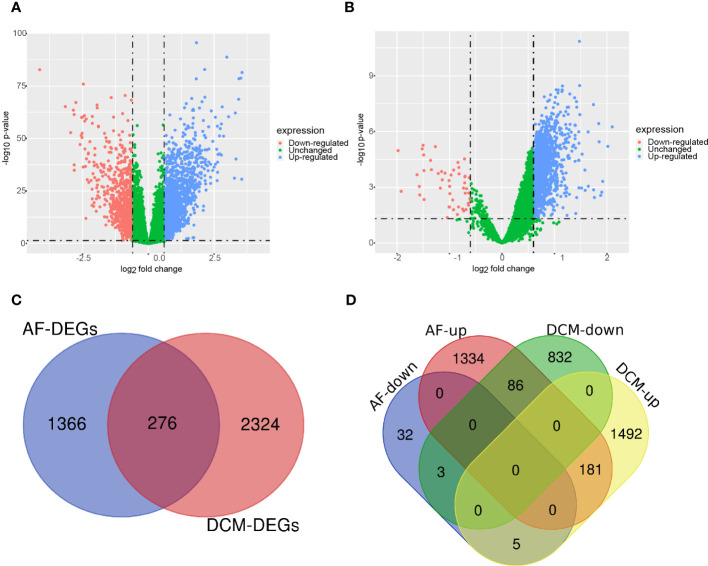
Volcano diagram and Venn diagram. **(A)** The volcano map of GSE141910 (DCM). **(B)** The volcano map of GSE41177 and GSE79768 (AF). Downregulated genes are marked in light red; upregulated genes are marked in light blue. **(C)** The DCM and AF datasets showed an overlap of 276 DEGs. **(D)** 184 DEGs were obtained after excluding genes with opposite expression trends.

The research flowchart of this bioinformatics analysis is shown in **Figure 1**. We merged the GSE41177 and GSE79768 (AF) gene expression datasets and then removed inter-batch differences using the R package “sva”. We utilized the R package “limma” to identify DEGs between controls and disease patients with DCM or AF. The results presented that 921 genes were significantly down-regulated and 1678 genes were significantly up-regulated in DCM (**Figure 2A**), whereas 40 genes were down-regulated, and 1601 genes were up-regulated in AF (**Figure 2B**). After genes with opposite expression trends were removed, we obtained 184 DEGs ultimately (**Figure 2C, D**, **Supplementary Table S1**).

### Functional enrichment analysis of DEGs

**Figure 3:**
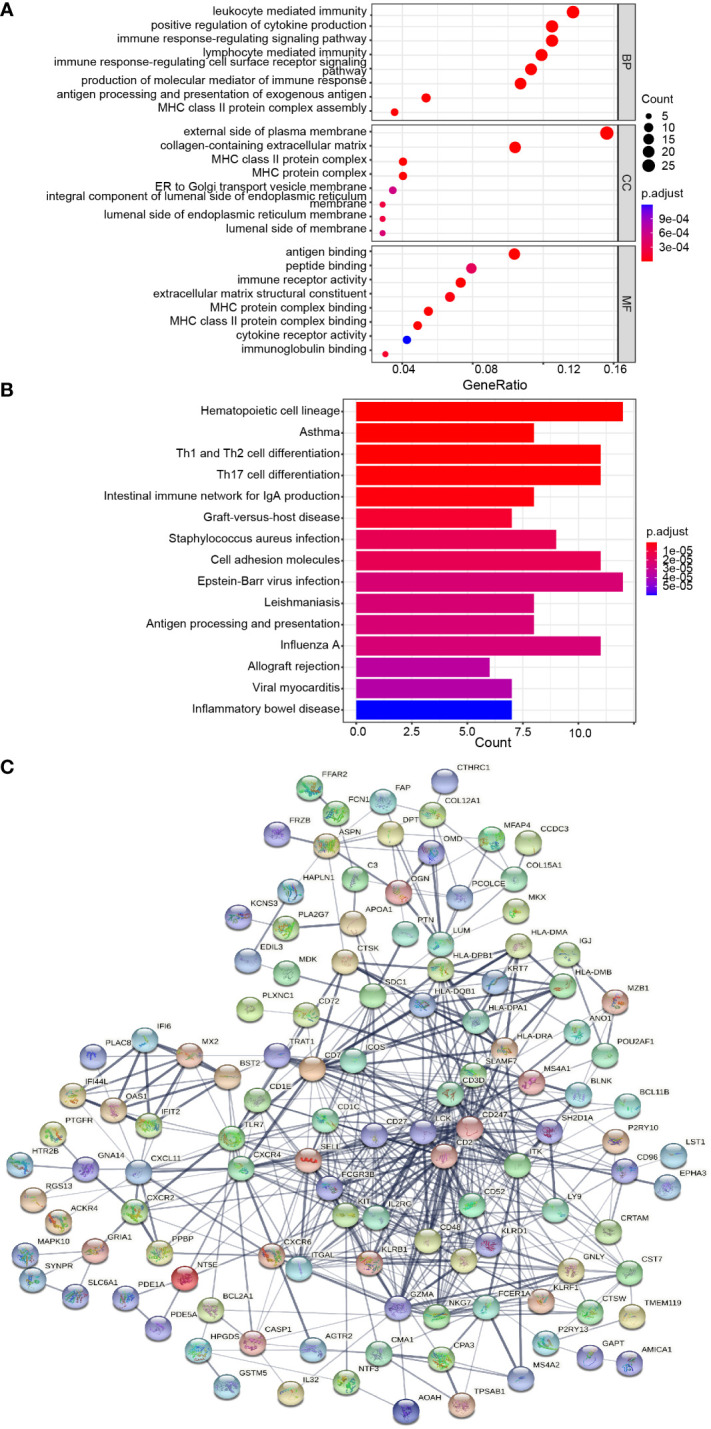
Protein-protein interaction (PPI) network and common DEGs enrichment analysis results. **(A, B)** The enrichment analysis results of GO and KEGG Pathway. Adjusted *P*-value < 0.05 was considered significant. **(C)** PPI network of the DEGs (score > 0.4) contained 170 nodes and 518 interaction pairs.

GO and KEGG enrichment analyses were conducted to analyze the biological functions involved in the 184 DEGs. As shown in **Figure 3A**, GO analysis results indicated that the biological pathways of these 184 DEGs were significantly enriched in leukocyte-mediated immunity, immune response-regulating pathway, positive regulation of cytokine production, and lymphocyte-mediated immunity. As shown in **Figure 3B**, KEGG enrichment showed several biological pathways, including Th1 and Th2 cell differentiation, Th17 cell differentiation, and Intestinal immune network for IgA production, etc. Collectively, the function of 184 DEGs was markedly associated with immune response.

### PPI network construction and module analysis

**Figure 4:**
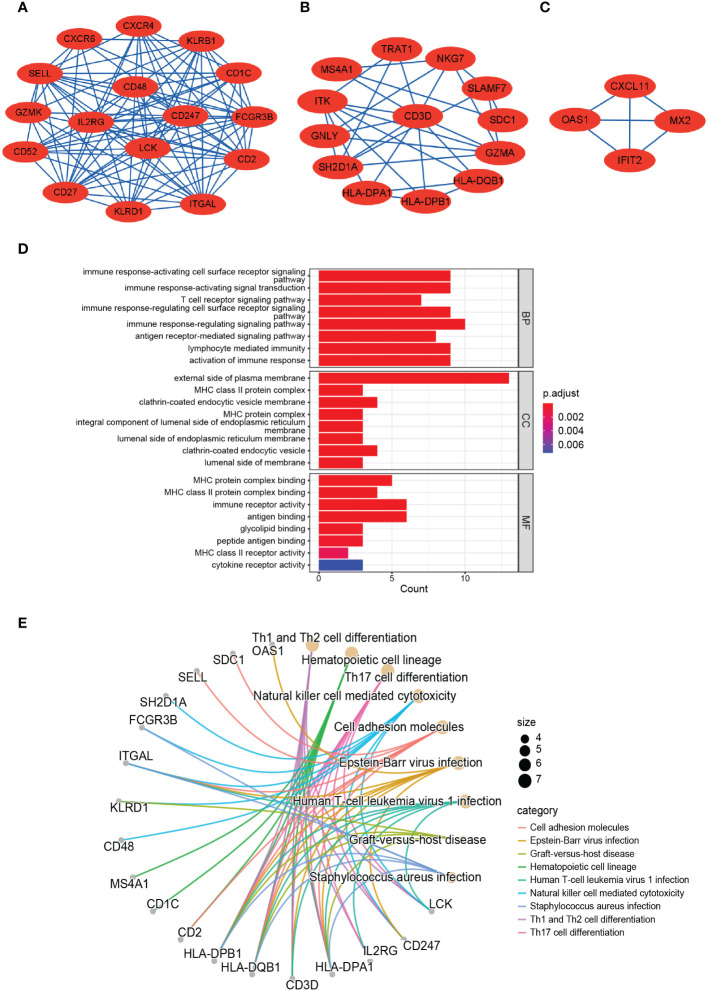
TOP 3 closely connected gene modules and enrichment analysis of the modular genes. **(A–C)** Three significant gene clustering modules. **(D)** Barplot of GO enrichment analysis of the modular genes. **(E)** Circular path diagram of KEGG enrichment analysis of the modular genes.

The PPI network of the 184 DEGs was established through STRING database (score > 0.4), containing 170 nodes and 518 interaction pairs (**Figure 3C**). TOP three key gene modules, which included 33 common DEGs (**Supplementary Table S2**) and 132 interaction pairs (**Figures 4A–C**), were obtained using the MCODE plugin of Cytoscape. GO analysis presented that these genes were related to immune response, antigen processing, and cell killing (**Figure 4D**). KEGG Pathway enrichment analysis revealed that they were mainly enriched in the Th1 and Th2 cell differentiation pathway, Th17 cell differentiation pathway, and Natural killer (NK) cell-mediated cytotoxicity (**Figure 4E**).

### Identification and analysis of hub genes

**Figure 5:**
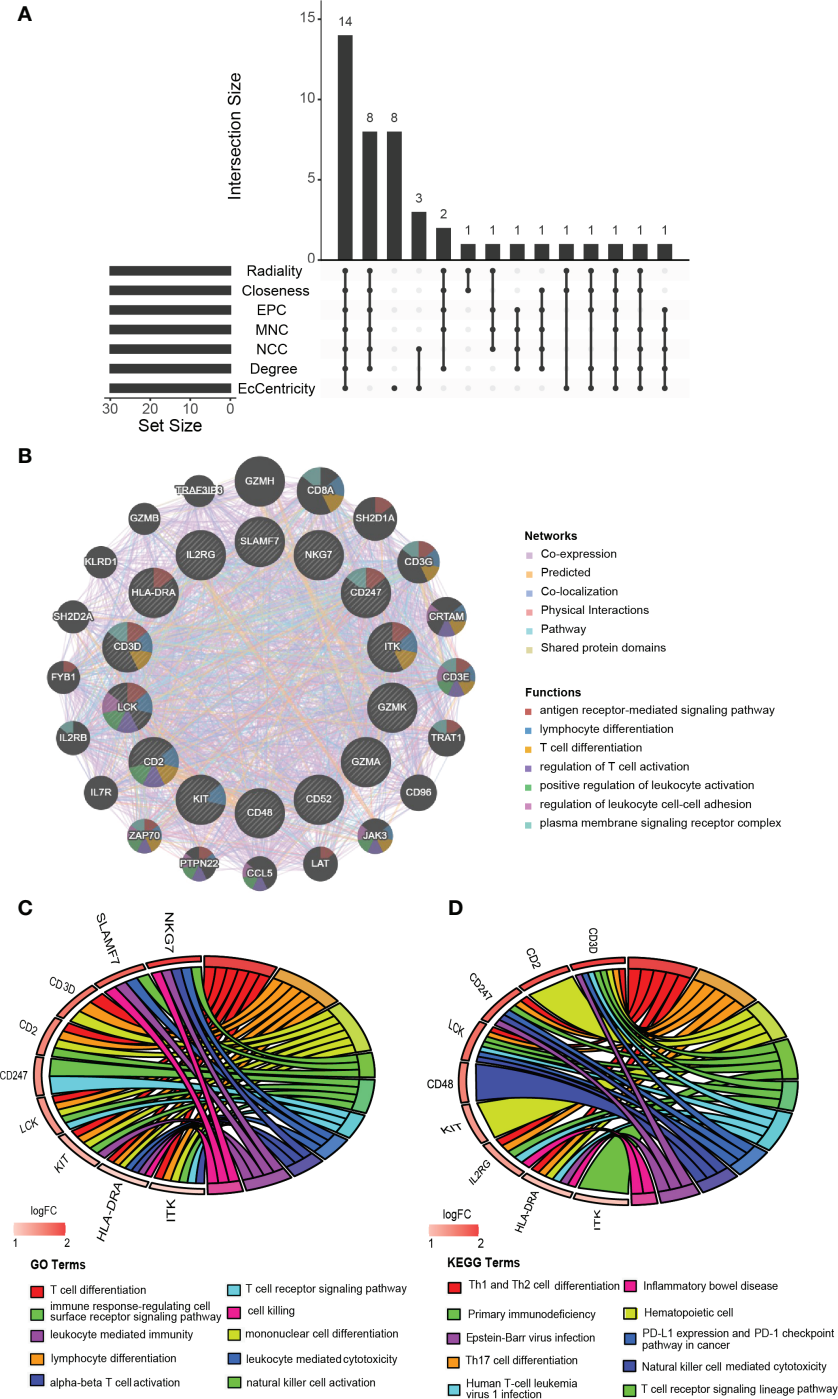
Identification, co-expression network and enrichment analysis of the hub genes. **(A)** The Venn diagram showed that seven algorithms have screened out 14 overlapping hub genes. **(B)** Hub genes and their co-expression genes were analyzed *via* GeneMANIA. **(C, D)** GO and KEGG enrichment analysis of the hub genes.

We acquired the top 30 hub genes from 184 DEGs through the seven algorithms of plugin cytoHubba (**Supplementary Table S3**). We screened 14 hub genes (*LCK*, *HLA-DRA*, *ITK*, *CD2*, *CD52*, *GZMK*, *CD48*, *SLAMF7*, *CD247*, *KIT*, *IL2RG*, *NKG7*, *CD3D*, and *GZMA*) by R package “UpSet” and visualized them with the histogram in **Figure 5A**. We constructed the co-expression network and predicted the functions of these genes through the GeneMANIA database. These 14 hub genes presented a complex PPI network with a co-expression of 63.41%, co-localization of 6.57%, physical interactions of 4.77%, predicted of 18.03%, and pathway of 4.50% (**Figure 5B**). As revealed by GO analysis, these genes played a significant role in T cell differentiation, lymphocyte differentiation, mononuclear cell differentiation, NK cell activation, immune response, and leukocyte-mediated cytotoxicity (**Figure 5C**). In addition, KEGG enrichment analysis revealed that they were mainly enriched in the Th1 and Th2 cell differentiation pathway, Th17 cell differentiation pathway, T cell receptor signaling pathway, and NK cell-mediated cytotoxicity pathway (**Figure 5D**).

### Screening of hub immune-related genes and analyses of immune infiltration in DCM and AF

**Figure 6:**
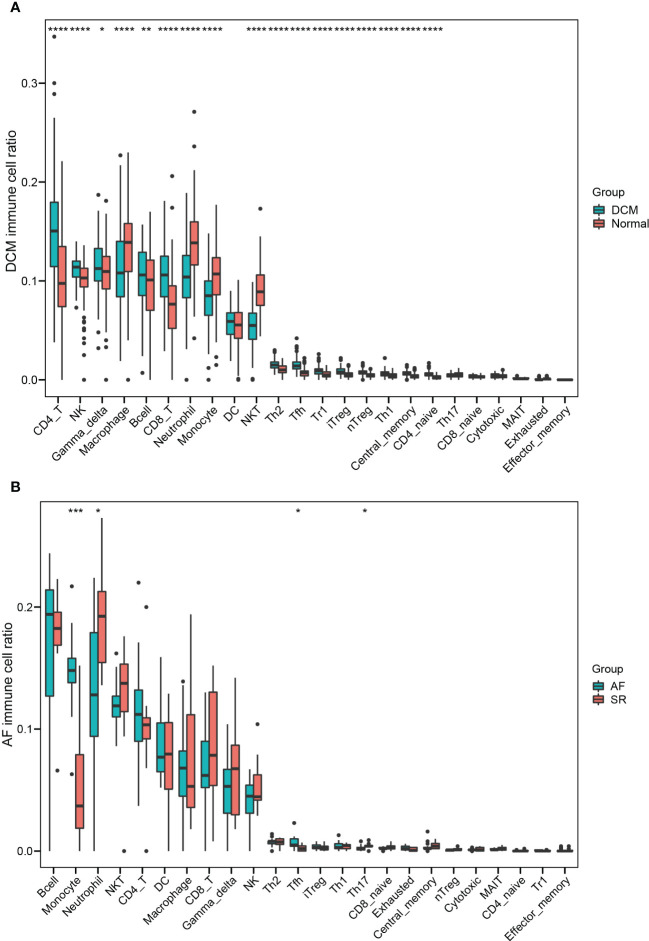
Identifying the significantly different infiltrates of immune cells in DCM and AF. **(A)** Boxplot of immune cell ratio in DCM. **(B)** Boxplot of immune cell ratio in AF. Wilcoxon test: * *P* < 0.05, ** *P* < 0.01, *** *P* < 0.001, **** *P* < 0.0001.

**Figure 7:**
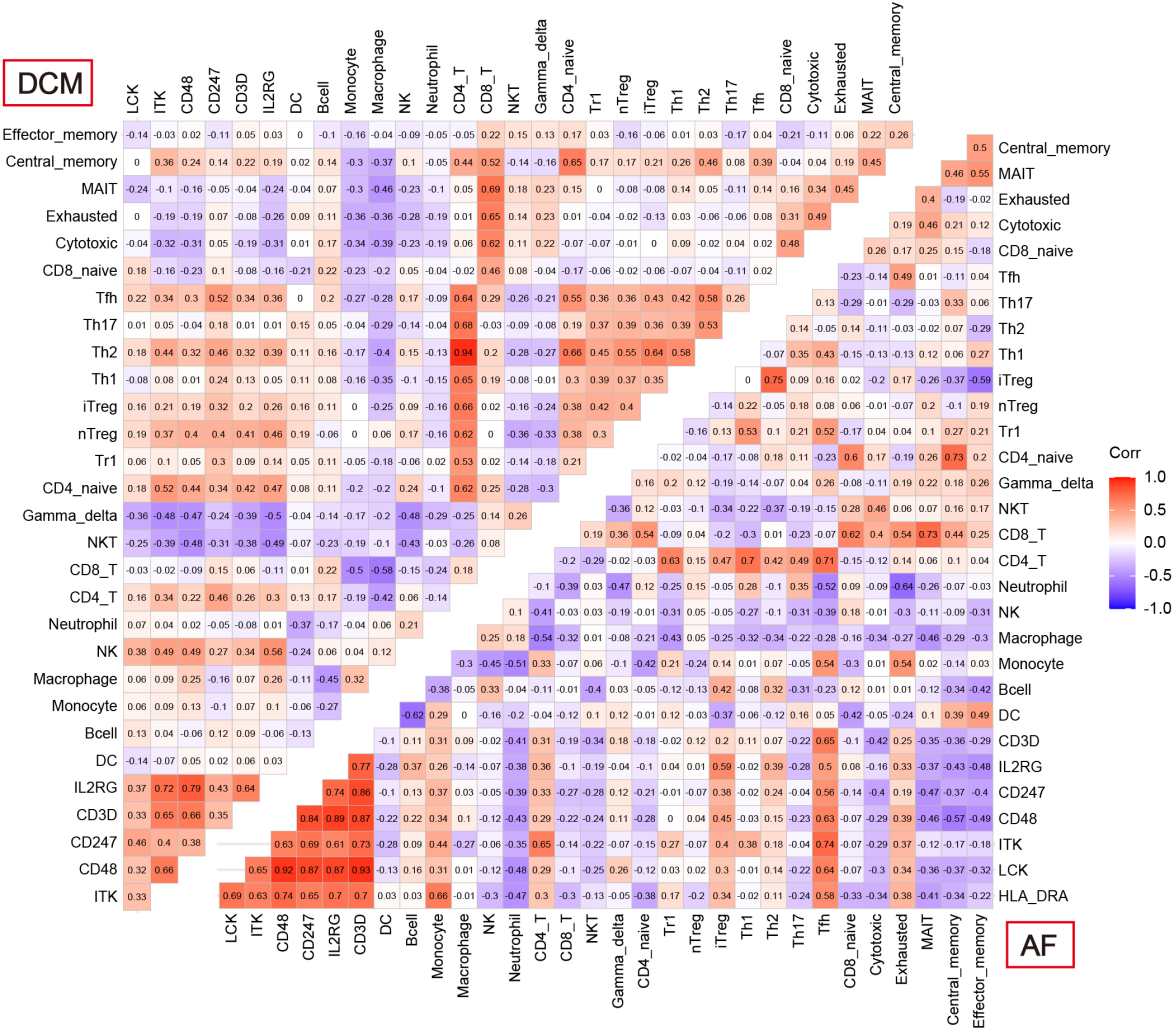
Correlation between 7 hub immune-related genes and 24 differential immune cells in DCM and AF.

Enrichment analysis showed that multiple immune-related biological functions were simultaneously abnormal in DCM and AF. Therefore, we considered that the pathogenesis of DCM combined with AF was related to immune disorders. Subsequently, we identified hub immune-related genes and analyzed immune cell infiltration in patients with DCM and AF. 1793 immune-related genes were obtained from the ImmPort database (**Supplementary Table S4**), among which 7 common genes (*HLA-DRA*, *LCK*, *ITK*, *CD48*, *CD247*, *CD3D*, and *IL2RG*) intersected with hub genes in the immune-related gene list. These 7 immune-related genes were significantly elevated in DCM and AF patients compared with controls. We employed ImmuCellAI algorithms to evaluate the immune infiltration patterns in DCM and AF. The abundance of Macrophage, Neutrophil, Monocyte, and NKT cells was markedly decreased in DCM patients compared with controls, whereas the abundance of CD4^+^T cells, NK cells, Gamma_delta cells, B cells, CD8^+^T cells, Th2 cells, Tfh cells, Tr1 cells, iTreg cells, nTreg cells, Th1 cells, entral_memory cells, and CD4^+^naive cells was markedly up-regulated (**Figure 6A**). The abundance of Neutrophil and Th17 cells was significantly down-regulated in patients with AF compared with controls, whereas the abundance of Monocyte and Tfh cells was significantly up-regulated (**Figure 6B**). These results suggest that patients with DCM and AF share some of the same regulated immune cells. Moreover, we investigated the relevance between the expression levels of hub immune-related genes and immune cell abundance in DCM and AF patients. The results indicated that the identified hub immune-related genes were significantly associated with immune cells simultaneously dysregulated in DCM and AF patients. For example, the abundance of Tfh cells was significantly reduced in both DCM and AF patients, and hub immune-related common genes were predominantly favorably correlated with Tfh cells, CD4^+^T cells, iTreg cells, and Th2 cells infiltration and primarily negatively correlated with Neutrophil, Th17 cells, and NKT cells infiltration in DCM and AF patients (**Figure 7**).

### Validation of hub immune-related expression and immune infiltration

**Figure 8:**
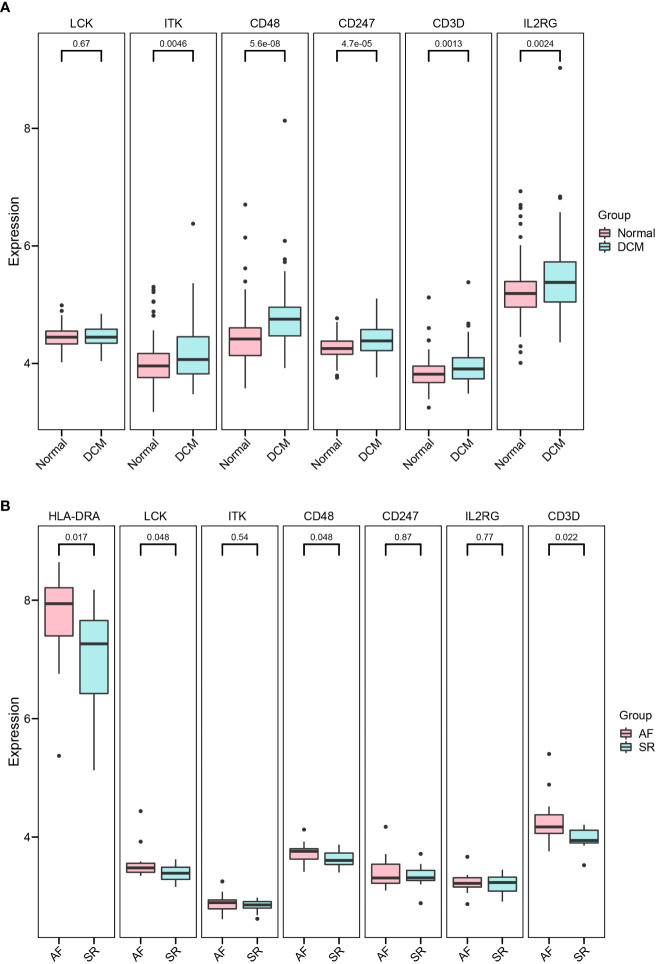
Validation of hub immune-related genes. **(A, B)** Detailed expressions of 7 hub immune-related genes were validated in datasets GSE57338 (DCM), GSE115574, and GSE31821(AF).

**Figure 9:**
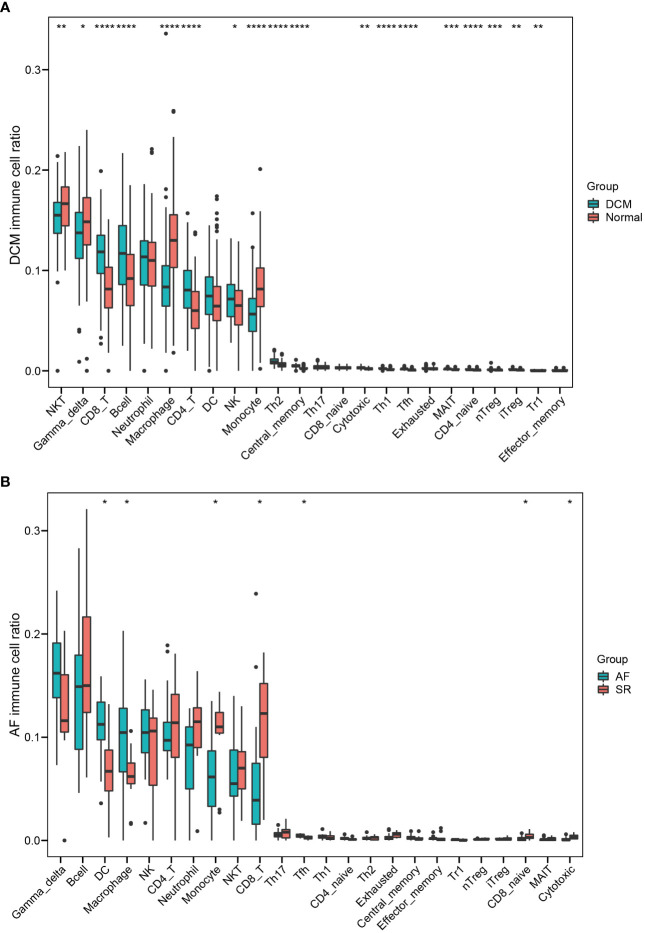
Validating the significantly different infiltrates of immune cells in DCM and AF. **(A)** Boxplot of immune cell ratio in DCM. **(B)** Boxplot of immune cell ratio in AF. Wilcoxon test: * *P* < 0.05, ** *P* < 0.01, *** *P* < 0.001, **** *P* < 0.0001.

**Figure 10:**
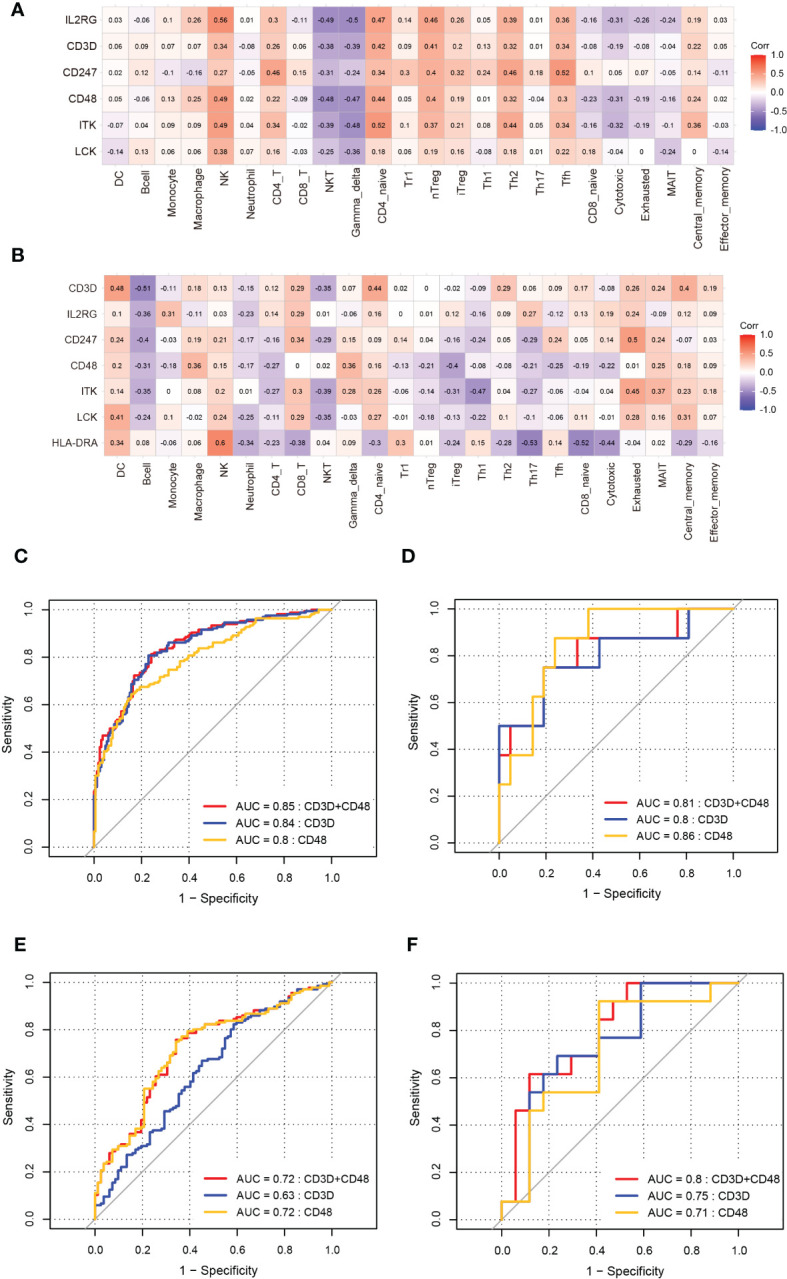
Validation of correlation between hub immune-related genes and 24 immune cells and ROC analysis of biomarkers in DCM and AF. **(A, B)** The correlation between hub immune-related genes and 24 immune cells in datasets GSE57338 (DCM), GSE115574, and GSE31821(AF). **(C)** ROC analysis of each hub immune-related genes in the training dataset (DCM). **(D)** ROC analysis of each hub immune-related genes in the training dataset (AF). **(E)**The GSE57338 dataset was used to validate the diagnostic effectiveness of the biomarkers for DCM by ROC analysis. **(F)**The GSE115574 and GSE31821datasets were used to validate the diagnostic effectiveness of the biomarkers for AF by ROC analysis.

Independent datasets (GSE57338, GSE115574, and GSE31821) were used to validate the results of hub immune-related expression and immune infiltration. In the same way, the expression data of 7 hub immune-related genes were extracted from validation datasets, and two of them were in accordance with the tendency in the training datasets, including *CD48* and *CD3D* (**Figures 8A, B**). Immune cell infiltration analysis presented that Tfh cells were also significantly increased in DCM and AF patients in the validation datasets (**Figures 9A, B**). Overall, the results of the training datasets and the validation datasets were consistent (**Figures 10A, B**). AF mediated cardiomyopathy (also termed tachycardia mediated cardiomyopathy) is a newly described syndrome in which AF is the primary cause of HF which can be reversible after successful treatment of AF. The relationship between these two from a mechanistic perspective is largely unknown. We then obtained dataset (GSE86569) of AF without HF and AF with HF to analyze the immune infiltration patterns and verify the expression of immune-related genes (*CD48* and *CD3D*). We found a significant decrease in the proportion of Exhausted cells and B cells and an increase in the proportion of Central_memory cells, Th2 cells, CD4_naive cells, iTreg cells, Tr1 cells, and nTreg cells in the cardiac tissue samples of AF patients with HF compared with AF patients without HF. These results showed that the pattern of immune infiltration in the cardiac tissue of patients with AF and HF was mainly characterized by multiple T cells disorders. *CD48* was significantly increased in the cardiac tissues of AF patients with HF compared with AF patients without HF. These results are in partial agreement with our results in the DCM and AF analyses (**Supplementary File**).

### ROC analysis of biomarkers

The ROC analysis was applied to predict the diagnostic efficacy of the hub immune-related genes (*CD48* and *CD3D*) for DCM and AF in the training and validation datasets. In the DCM training dataset, the AUC values of *CD48*, *CD3D*, and the two together were 0.8, 0.84, and 0.85, respectively (**Figure 10C**). In the AF training datasets, the AUC values of *CD48*, *CD3D*, and the two together were 0.86, 0.8, and 0.81, respectively (**Figure 10D**). In the DCM validation dataset, the AUC values of *CD48*, *CD3D*, and the two together were 0.72, 0.63, and 0.72, respectively (**Figure 10E**). In the AF validation dataset, the AUC values of *CD48*, *CD3D*, and the two together were 0.71, 0.75, and 0.8, respectively (**Figure 10F**). Therefore, *CD48*+*CD3D* could diagnose DCM and AF with excellent specificity and sensitivity, respectively, showing excellent diagnostic value.

## Discussion

DCM complicated with AF is one of the common causes of heart failure and sudden death (4). Nevertheless, the etiology of DCM complicated with AF has not been fully elucidated. In this bioinformatics research, we token advantage of comprehensive and effective bioinformatics analysis methods to investigate the hub genes of DCM complicated with AF, analyze immune infiltration patterns in the DCM and AF, and explore the possible molecular mechanism of DCM complicated with AF. In addition, we assessed the diagnostic efficacy of the biomarkers by ROC analysis.

DCM and AF might have overlapping pathogenic pathways. In this research, we identified 184 common DEGs between them, of which 14 were defined as hub genes, including *LCK*, *HLA-DRA*, *ITK*, *CD2*, *CD52*, *GZMK*, *CD48*, *SLAMF7*, *CD247*, *KIT*, *IL2RG*, *NKG7*, *CD3D*, and *GZMA*. Some of the above hub genes have been reported to play significant roles in DCM and AF. For example, T-cell genes *CD3D* significantly increased diagnostic thresholds of DCM (15). GO and KEGG Pathway enrichment analysis indicated that these hub genes were mainly involved in immune response pathways, especially in Th1 cell differentiation, Th2 cell differentiation, and Th17 cell differentiation. Inflammation and immune response are significant pathogenesis of DCM and AF (1, 16). In the pathogenesis of viral myocarditis, the Th2 immune response induces ventricular remodeling and promotes the progression of myocarditis to DCM and heart failure, whereas the Th1 response alleviates viral myocarditis but increases acute myocardial inflammation by inhibiting Th2 responses (17). Th17 cells increase tissue inflammation and promote autoimmune activation by secreting large amounts of proinflammatory cytokines, while Treg cells can inhibit the body’s immune response and active tolerance to self-antigens through intercellular contact and secretion of inhibitory cytokines in various immune cell subsets, so as to avoid the occurrence of autoimmune diseases (18). Thus, the imbalance of Th17/Treg cells in the body has been proposed as a major mechanism of autoimmune diseases (18, 19). Notably, our study found that T cell differentiation plays a significant role in the pathogenesis of DCM complicated with AF. Nevertheless, there are few studies on the role of T cell-induced immune responses in AF. At present, a matched case-control study showed that elevated plasma levels of Th17-related cytokines were related to an increased risk of AF, suggesting that Th17-related cytokines may participate in the pathogenesis of AF (20). Further studies are required to explore the role and mechanism of the immune response in AF. By reason of the foregoing, these results suggested that inflammatory response and immune disorders regulated by hub genes play a significant role in the co-pathogenesis of DCM and AF.

In the present study, we obtained a list of 1796 immune-related genes from the Immport database and intersected them with hub genes to obtain 7 hub immune-related genes, including *HLA-DRA*, *LCK*, *ITK*, *CD48*, *CD3D*, *CD247*, and *IL2RG*. These 7 immune-related genes were significantly elevated in DCM and AF patients compared with controls. *HLA-DRA* is an MHC II antigen, which plays a significant role in cell-cell interactions in immune response (21). *LCK*, which is an Src kinase family member, plays an essential role in T cell receptor (TCR) signaling and T cell development and activation (22). *CD48* is the ligand for *CD2* that facilitates interaction between activated lymphocytes (23). *CD3D* is part of the TCR-CD3 complex present on the T-lymphocyte cell surface that is mainly involved in T cell development and signal transduction (24). *ITK* is a tyrosine kinase that plays a significant role in the regulation of proximal TCR signaling (25). *CD247*, which encodes the *CD3* zeta chain, is mainly involved in the assembly of the TCR complex and signal transduction after antigen activation (26). *IL2RG* is a cytokine receptor common subunit gamma, which is mainly involved in T cell and NK cell development (27). Collectively, these hub immune-related genes are closely related to T cell function and immune response.

Subsequently, we applied ImmuCellAI to analyze immune infiltration between different tissues and investigated the correlation between the abundance of immune cells with the expression levels of hub immune-related genes. The research results suggested that Neutrophil and Tfh cells as hub immune cells were simultaneously dysregulated in DCM and AF patients’ cardiac tissues. The infiltration level of Tfh cells was significantly up-regulated in DCM and AF. Furthermore, the Spearman correlation between immune cells abundance and hub immune-related genes showed that 7 significantly upregulated immune-related genes (*HLA-DRA*, *LCK*, *ITK*, *CD48*, *CD247*, *CD3D*, and *IL2RG*) were predominantly favorably correlated with Tfh cells, CD4^+^T cells, iTreg cells, and Th2 cells infiltrations and primarily negatively correlated with Neutrophil cells, Th17 cells, and NKT cells infiltrations in DCM and AF patients. Tfh cells are a specialized subtype of CD4^+^T cells, which provide the relevant B-T cell interactions and cytokines to promote germinal center (GC) formation, promote GC B cell differentiation into memory B cells or plasma cells, and drive the development of high-affinity antibodies (28). Deregulation of Tfh cells’ activity promotes the production of pathogenic autoantibodies and plays an important role in promoting autoimmune diseases (29). Many studies have shown that Tfh cells disorders are related to multiple autoimmune diseases, including rheumatoid arthritis, systemic lupus erythematosus, Sjogren’s syndrome, vasculitis, systemic sclerosis, and so on (29, 30). Our study showed that Tfh cell disorder caused by hub immune-related genes might be the common pathogenesis of DCM and AF. In the validation datasets, the expression levels of *CD3D* and *CD48* were consistent with the trend in the training datasets, so we selected *CD3D* and *CD48* as biomarkers for the diagnosis of DCM and AF. AF is a common persistent arrhythmia in patients with DCM, which is often complicated with each other. Cardiomyopathy induced by AF can contribute to the progression of HF. We found that the immune infiltration patterns in the cardiac tissues of AF patients with HF were mainly manifested as the disorder in the proportion of multiple T cells, which may be related to the dysregulated expression of immune-related genes. These findings were partially consistent with our study in DCM and AF. In the ROC analysis, we found that *CD48*+*CD3D* could diagnose DCM and AF with excellent specificity and sensitivity in the training datasets and validation datasets, respectively. The above findings have significant implications for the field of cardiovascular disease, suggesting that future researches focus on explaining the functional roles of the identified hub immune-related genes and the corresponding immune cell disorders mechanism in DCM complicated with AF.

The study has shown that absence of recurrent atrial tachyarrhythmia after ablation of AF in patients with DCM is associated with improvement in HF within 3 years (but not more than 3 years) after ablation of AF (31). It can be concluded that the recovery of sinus rhythm is very important for the improvement of prognosis in patients with DCM and AF. However, there is no evidence that the disorder of immune-related genes in patients with DCM and AF is related to heart rate, which will be the direction of future research. We found that the immune cell disorders mediated by hub immune-related genes may be the common pathological mechanism of DCM and AF. In clinical practice, genetic testing should be carried out in patients with early DCM. If genetic testing suggests an increase in hub immune-related genes, it means that the patients with DCM have a higher risk of AF. It is recommended that patients with regular electrocardiogram and 24-hours Holter detection, timely diagnosis of AF and corresponding blocking treatment are helpful to improve the prognosis of patients with DCM.

Some studies have identified the core pathogenic genes of DCM (32) and AF (33), respectively. However, at present, fewer studies have elucidated the common pathogenesis between them. Due to the high comorbidity rate between DCM and AF, we identified for the first time the common DEGs and hub immune-related genes in DCM and AF, and analyzed immune infiltration between them, which is helpful to further elucidate the common pathogenesis of DCM and AF. Of course, some limitations should be noted in our study. First of all, this is a bioinformatics study that requires confirmatory experiments to demonstrate our results. Secondly, the study lacks clinically relevant information, including cardiac function and inflammatory biomarkers, etc. Finally, we lack experimental data on the regulatory mechanisms of dysregulated hub immune-related genes and immune cells. Thus, further studies are required to investigate the role and regulatory mechanism of the immune-related genes induced immune cell disorder in DCM complicated with AF.

## Conclusion

In summary, we applied the bioinformatics analysis method to identify hub immune-related genes concurrently involved in DCM and AF, explore their association with immune infiltration, and evaluate the diagnostic value of hub immune-related genes in DCM and AF. The above findings lay a foundation for future studies to elucidate the pathogenesis of immune responses in DCM complicated with AF.

## Data availability statement

The original contributions presented in the study are included in the article/**Supplementary Material**. Further inquiries can be directed to the corresponding authors.

## Author contributions

TG, Yan W and ZW devised the concept and supervised the study. TG analyzed the data and drafted the article. TG, JH, AA, WL, CL, SL, Ya W, and QX contributed to reviewing the article. All authors contributed to the manuscript revision and approved the submitted version.

## References

[1] Schultheiss HP Fairweather D Caforio ALP Escher F Hershberger RE Lipshultz SE. Dilated cardiomyopathy. Nat Rev Dis Primers (2019) 5(1):32. doi: 10.1038/s41572-019-0084-1 31073128 PMC7096917

[2] Maron BJ Towbin JA Thiene G Antzelevitch C Corrado D Arnett D. Contemporary definitions and classification of the cardiomyopathies: An American heart association scientific statement from the council on clinical cardiology, heart failure and transplantation committee; quality of care and outcomes research and functional genomics and translational biology interdisciplinary working groups; and council on epidemiology and prevention. Circulation (2006) 113(14):1807–16. doi: 10.1161/circulationaha.106.174287 16567565

[3] Verma A Kalman JM Callans DJ. Treatment of patients with atrial fibrillation and heart failure with reduced ejection fraction. Circulation (2017) 135(16):1547–63. doi: 10.1161/circulationaha.116.026054 28416525

[4] Carlisle MA Fudim M DeVore AD Piccini JP. Heart failure and atrial fibrillation, like fire and fury. JACC Heart Fail (2019) 7(6):447–56. doi: 10.1016/j.jchf.2019.03.005 31146871

[5] Lardizabal JA Deedwania PC. Atrial fibrillation in heart failure. Med Clin North Am (2012) 96(5):987–1000. doi: 10.1016/j.mcna.2012.07.007 22980060

[6] Edgar R Domrachev M Lash AE. Gene expression omnibus: Ncbi gene expression and hybridization array data repository. Nucleic Acids Res (2002) 30(1):207–10. doi: 10.1093/nar/30.1.207 PMC99122 11752295

[7] Leek JT Johnson WE Parker HS Jaffe AE Storey JD. The sva package for removing batch effects and other unwanted variation in high-throughput experiments. Bioinformatics (2012) 28(6):882–3. doi: 10.1093/bioinformatics/bts034 PMC3307112 22257669

[8] Ritchie ME Phipson B Wu D Hu Y Law CW Shi W. Limma powers differential expression analyses for rna-sequencing and microarray studies. Nucleic Acids Res (2015) 43(7):e47. doi: 10.1093/nar/gkv007 25605792 PMC4402510

[9] Yu G Wang LG Han Y He QY. Clusterprofiler: An r package for comparing biological themes among gene clusters. Omics (2012) 16(5):284–7. doi: 10.1089/omi.2011.0118 PMC3339379 22455463

[10] Franceschini A Szklarczyk D Frankild S Kuhn M Simonovic M Roth A. String V9.1: Protein-protein interaction networks, with increased coverage and integration. Nucleic Acids Res (2013) 41(Database issue):D808–15. doi: 10.1093/nar/gks1094 PMC3531103 23203871

[11] Smoot ME Ono K Ruscheinski J Wang PL Ideker T. Cytoscape 2.8: New features for data integration and network visualization. Bioinformatics (2011) 27(3):431–2. doi: 10.1093/bioinformatics/btq675 PMC3031041 21149340

[12] Warde-Farley D Donaldson SL Comes O Zuberi K Badrawi R Chao P. The genemania prediction server: Biological network integration for gene prioritization and predicting gene function. Nucleic Acids Res (2010) 38(Web Server issue):W214–20. doi: 10.1093/nar/gkq537 PMC2896186 20576703

[13] Bhattacharya S Dunn P Thomas CG Smith B Schaefer H Chen J. Immport, toward repurposing of open access immunological assay data for translational and clinical research. Sci Data (2018) 5:180015. doi: 10.1038/sdata.2018.15 29485622 PMC5827693

[14] Miao YR Zhang Q Lei Q Luo M Xie GY Wang H. Immucellai: A unique method for comprehensive T-cell subsets abundance prediction and its application in cancer immunotherapy. Adv Sci (Weinh) (2020) 7(7):1902880. doi: 10.1002/advs.201902880 32274301 PMC7141005

[15] Noutsias M Rohde M Göldner K Block A Blunert K Hemaidan L. Expression of functional T-cell markers and T-cell receptor vbeta repertoire in endomyocardial biopsies from patients presenting with acute myocarditis and dilated cardiomyopathy. Eur J Heart failure (2011) 13(6):611–8. doi: 10.1093/eurjhf/hfr014 21422001

[16] Hu YF Chen YJ Lin YJ Chen SA. Inflammation and the pathogenesis of atrial fibrillation. Nat Rev Cardiol (2015) 12(4):230–43. doi: 10.1038/nrcardio.2015.2 25622848

[17] Zheng SY Dong JZ. Role of toll-like receptors and Th responses in viral myocarditis. Front Immunol (2022) 13:843891. doi: 10.3389/fimmu.2022.843891 35514979 PMC9062100

[18] Lee GR. The balance of Th17 versus treg cells in autoimmunity. Int J Mol Sci (2018) 19(3):730. doi: 10.3390/ijms19030730 29510522 PMC5877591

[19] Li J Wang L Wang S Zhu H Ye P Xie A. The Treg/Th17 imbalance in patients with idiopathic dilated cardiomyopathy. Scand J Immunol (2010) 71(4):298–303. doi: 10.1111/j.1365-3083.2010.02374.x 20384874

[20] Wu N Xu B Liu Y Chen X Tang H Wu L. Elevated plasma levels of Th17-related cytokines are associated with increased risk of atrial fibrillation. Sci Rep (2016) 6:26543. doi: 10.1038/srep26543 27198976 PMC4873818

[21] Mulder DJ Pooni A Mak N Hurlbut DJ Basta S Justinich CJ. Antigen presentation and mhc class ii expression by human esophageal epithelial cells: Role in eosinophilic esophagitis. Am J Pathol (2011) 178(2):744–53. doi: 10.1016/j.ajpath.2010.10.027 PMC3069880 21281807

[22] Kumar Singh P Kashyap A Silakari O. Exploration of the therapeutic aspects of lck: A kinase target in inflammatory mediated pathological conditions. BioMed Pharmacother (2018) 108:1565–71. doi: 10.1016/j.biopha.2018.10.002 30372858

[23] Tufa DM Yingst AM Trahan GD Shank T Jones D Shim S. Human innate lymphoid cell precursors express Cd48 that modulates ilc differentiation through 2b4 signaling. Sci Immunol (2020) 5(53):eaay4218. doi: 10.1126/sciimmunol.aay4218 33219153 PMC8294935

[24] Wang Q Li P Wu W. A systematic analysis of immune genes and overall survival in cancer patients. BMC Cancer (2019) 19(1):1225. doi: 10.1186/s12885-019-6414-6 31842801 PMC6915928

[25] Weeks S Harris R Karimi M. Targeting itk signaling for T cell-mediated diseases. iScience (2021) 24(8):102842. doi: 10.1016/j.isci.2021.102842 34368657 PMC8326193

[26] Rudemiller N Lund H Jacob HJ Geurts AM Mattson DL. Cd247 modulates blood pressure by altering T-lymphocyte infiltration in the kidney. Hypertension (Dallas Tex: 1979) (2014) 63(3):559–64. doi: 10.1161/hypertensionaha.113.02191 PMC3945169 24343121

[27] Lin JX Leonard WJ. The common cytokine receptor Γ chain family of cytokines. Cold Spring Harb Perspect Biol (2018) 10(9):a028449. doi: 10.1101/cshperspect.a028449 29038115 PMC6120701

[28] Crotty S. Follicular helper Cd4 T cells (Tfh). Annu Rev Immunol (2011) 29:621–63. doi: 10.1146/annurev-immunol-031210-101400 21314428

[29] Gensous N Charrier M Duluc D Contin-Bordes C Truchetet ME Lazaro E. T Follicular helper cells in autoimmune disorders. Front Immunol (2018) 9:1637. doi: 10.3389/fimmu.2018.01637 30065726 PMC6056609

[30] Crotty ST. Follicular helper cell biology: A decade of discovery and diseases. immunity, Vol. 50. (2019) 50(5):1132–48. doi: 10.1016/j.immuni.2019.04.011. PMC6532429 31117010

[31] Zhao L Xu K Jiang W Zhou L Wang Y Zhang X. Long-term outcomes of catheter ablation of atrial fibrillation in dilated cardiomyopathy. Int J Cardiol (2015) 190:227–32. doi: 10.1016/j.ijcard.2015.04.186 25920033

[32] Zheng Y Liu Z Yang X Weng S Xu H Guo C. Exploring key genes to construct a diagnosis model of dilated cardiomyopathy. Front Cardiovasc Med (2022) 9:865096. doi: 10.3389/fcvm.2022.865096 35571180 PMC9091505

[33] Zhang J Huang X Wang X Gao Y Liu L Li Z. Identification of potential crucial genes in atrial fibrillation: A bioinformatic analysis. BMC Med Genomics (2020) 13(1):104. doi: 10.1186/s12920-020-00754-5 32682418 PMC7368672

