# Supplementary material for: Exploring the pathogenesis and immune infiltration in dilated cardiomyopathy complicated with atrial fibrillation by bioinformatics analysis
Source: Front Immunol. 2023 Jan 17;14:1049351. doi: 10.3389/fimmu.2023.1049351 (PMC9888493; doi:10.3389/fimmu.2023.1049351)
Supplement: Supplementary file 5 [file DataSheet_1.docx]

**Exploring the Pathogenesis and** **Immune Infiltration in Dilated Cardiomyopathy Complicated With Atrial Fibrillation by Bioinformatics Analysis**

Ting Gan, Jin Hu, ANWER KHALID OKAB Aledan, Wenhu Liu, Cui Li, Shuai Lu, Ya Wang, Qian Xu, Yan Wang, and Zhaohui Wang

**Supplementary Figure 1**…………………………………………………2


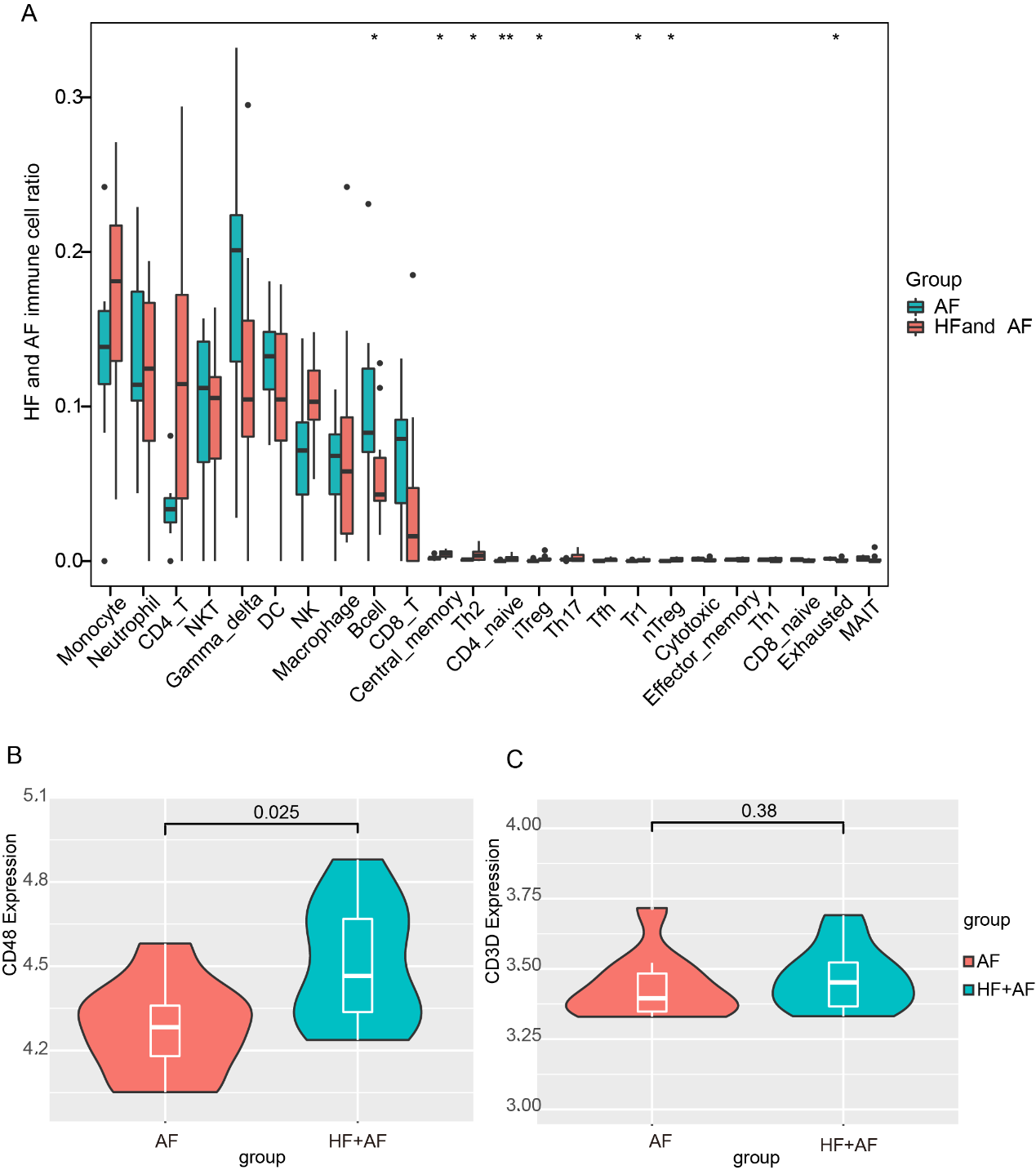


**Supplementary Figure 1** Validating the significantly different infiltrates of immune cells and the expressions of hub immune-related genes in AF and (or without) HF dataset (GSE86569). (A) Boxplot of immune cell ratio in AF and (or without) HF. (B) Boxplot of hub immune-related genes (*CD48* and *CD3D*) in AF and (or without) HF. Wilcoxon test: * *P* < 0.05, ** *P* < 0.01. AF: atrial fibrillation; HF: heart failure.
